# Development of TRPM8 Antagonists to Treat Chronic Pain and Migraine

**DOI:** 10.3390/ph10020037

**Published:** 2017-03-30

**Authors:** Andy D. Weyer, Sonya G. Lehto

**Affiliations:** 1 Pacific University School of Physical Therapy, Hillsboro, OR 97123, USA; andy.weyer@pacificu.edu; 2 One Amgen Center Dr, Thousand Oaks, CA 91320, USA

**Keywords:** transient receptor potential melastatin 8 (TRPM*), pain, menthol, cold hyperalgesia, cold analgesia, mechanical hyperalgesia, migraine

## Abstract

A review. Development of pharmaceutical antagonists of transient receptor potential melastatin 8 (TRPM8) have been pursued for the treatment of chronic pain and migraine. This review focuses on the current state of this progress.

## 1. Introduction

Transient receptor potential melastatin 8 (TRPM8) is a non-selective cation channel encoded by the *TRPM8* gene, first characterized as a detector of cold [1,2]. TRPM8 is found on both Aδ and C fiber afferents, and in addition to activation by cold temperatures, TRPM8 is activated by a number of chemical agonists that are known to produce cool sensations such as menthol, icilin, and eucalyptol [3,4]. As a natural extension of these findings, much research over the past decade has been devoted to the role of TRPM8-expressing afferents in the complex interpretation of hot and cold temperatures. Furthermore, the role of TRPM8 in pain sensation has been debated; indeed, while a large body of research has supported a role for TRPM8 in reducing or limiting pain sensation under injury conditions, an equally large number of publications propose that TRPM8 actually exaggerates pain after injury. From a pharmaceutical perspective, this complicates whether specific agonists or antagonists of TRPM8 should be developed to treat different pain conditions. In this review, we summarize the literature concerning the contribution of TRPM8 to both analgesia and nociception, and provide an update on the current state of drug development involving this versatile protein.

## 2. The Role for TRPM8 in Mechanical and Heat Analgesia

Intimately tied to our understanding of a potential role for TRPM8 in promoting analgesia is the effect of one of its prime agonists, menthol. Menthol is a common component of topical creams that have long been used to reduce pain and provide a cooling sensation [5]. Although some data has indicated that menthol may activate a variety of other channels, including transient receptor potential Ankyrin 1 (TRPA1), gamma-aminobutyric acid (GABA), and voltage-gated calcium and sodium channels [6,7], more recent studies have demonstrated that the prime target of menthol is indeed TRPM8, as genetic deletion of this receptor in mice prevents responsiveness to menthol at both the behavioral and cellular levels [8].

In animal studies, menthol has been shown to block the mechanical and heat hyperalgesia caused by injection of inflammatory compounds such as Complete Freund’s Adjuvant (CFA) or the transient receptor potential vanilloid 1 (TRPV1) agonist capsaicin [7,9,10]. Furthermore, injection of the TRPM8 agonist icilin significantly reduced the colonic damage observed in two different mouse models of inflammatory bowel disease [11]. In support of these inflammatory studies, it has been shown that components of the “inflammatory soup” that develops after an injury can inhibit TRPM8. Andersson and colleagues reported that low pH inactivates TRPM8, making it less responsive to the TRPM8 agonist icilin and cold temperatures (but interestingly not menthol) [12]. Similarly, another study found that bradykinin, a key potentiator of pain and component of the inflammatory soup, reduces TRPM8 activity through the action of protein kinase C in both the periphery and at the central synapse in the dorsal horn [10].

Adding to the view that TRPM8 promotes analgesia following injury are data from studies in which TRPM8 is either genetically deleted or experimentally knocked down. Proudfoot and colleagues first demonstrated this concept in a chronic constriction injury (CCI) model. Experimental rats in this study exhibited significantly reduced heat and mechanical pain behaviors when topical icilin was applied to the paw, but this effect was completely reversed when TRPM8 expression was knocked down via intrathecal injection of antisense oligonucleotides [13]. In similar experiments utilizing the CCI model, cooling or applying menthol to the affected paw resulted in reduced hypersensitivity in response to mechanical stimuli [14,15], but this effect was not seen when TRPM8 was knocked out or when TRPM8-expressing afferents were ablated [14]. Likewise, one of the first studies to utilize TRPM8 knockout mice demonstrated that TRPM8 was responsible for the analgesia provided by a cold plate during the first phase of the formalin test [16], and later studies using mice deficient in TRPM8 showed that menthol was unable to exert its analgesic effects in models of inflammatory pain using capsaicin or CFA [7].

A number of studies in humans also point toward a role for TRPM8 in mediating analgesia. In a recent study, injections of the TRPA1 agonist cinnamaldehyde into the forearm resulted in significant pain and neurogenic flare; however, simultaneous injection of menthol resulted in lower pain ratings, elevated mechanical pain thresholds, and reduced neurogenic flare as compared to cinnamaldehyde alone [17]. Two case studies also demonstrate the analgesic role of TRPM8 in patients suffering from chronic neuropathic pain. One individual developed neuropathic pain after long-term dosing with the chemotherapeutic Bortezomib, which causes neuropathy in up to 35% of patients. This individual suffered from a severe burning sensation in his lower limbs and “lightning-like” sensations in his hands. However, topical application of a 0.5% menthol cream to his lower extremities in a stocking distribution and the lumbosacral region overlying the affected nerve roots resulted in a significant improvement in response to suprathreshold mechanical stimuli and overall pain ratings [18]. Similarly, in another case where a patient suffered from severe allodynia following a case of post-herpetic neuralgia, application of menthol oil in concentrations of 2 or 10% resulted in a significant abatement of symptoms [19]. Further proof of the analgesic effects through TRPM8 can be observed in the ability of menthol or eucalyptol (another TRPM8 agonist) to prevent the irritant effects of acrolein and other cigarette smoke components [20].

## 3. The Role of TRPM8 in Cold Hyperalgesia

Much data also suggests that TRPM8 plays a role in amplifying pain sensation after injury, especially in models of neuropathic pain. Hypersensitivity to cold is a common complaint of individuals with neuropathies, with 20–30% of individuals diagnosed with different types of neuropathies complaining of cold hyperalgesia and elevated cold pain thresholds [21,22,23]. Likewise, other studies report significant elevations in cold pain thresholds in patients treated with the chemotherapeutic oxaliplatin, indicating that innocuous temperatures had become painful for these individuals [23,24]. In addition to alterations in cold thresholds, these individuals also rated specific cold temperatures as 3–4 times more painful than at baseline.

The cold hypersensitivity following neuropathic injury observed in human subjects has been consistently paralleled in animal models of nerve injury, and has been further extended to identify a definitive role for TRPM8 in mediating this pain. For instance, oxaliplatin-induced neuropathies in mice cause cold hypersensitivity on the behavioral level and also result in an increased percentage of isolated sensory neurons that respond to cold temperatures [25,26]. When TRPM8 knockout mice are utilized for these same experiments, this cold hyperalgesia is absent, implicating TRPM8 as a critical player in this phenomenon [25]. At this time it is unclear whether TRPM8 expression increases after installation of oxaliplatin-induced neuropathy, with one study reporting increased mRNA expression [27] and another reporting no change in mRNA expression levels [25].

Another popular neuropathic model is the CCI, in which ligatures are tied around the sciatic nerve. This model has consistently been shown to cause cold hypersensitivity, and multiple studies have reported that the increased responsiveness to the acetone evaporative cooling test following CCI was significantly reduced when TRPM8 was genetically deleted or when TRPM8-expressing afferents were chemically ablated [15,26]. Similarly, another study has reported that knockdown of TRPM8 with antisense oligonucleotides results in reduced responsiveness to cold as compared to animals injected with the missense oligonucleotide [14]. Additionally, this study and others have reported an increase in the number of neurons expressing TRPM8 [14,28,29] and an increase in total amount of TRPM8 protein in the DRG following CCI surgery [14]. Functionally, an increased number of isolated sensory neurons are responsive to cold and menthol after CCI, and these responses are potentiated as compared to controls [28].

## 4. The Role of TRPM8 in Bladder Pain

Interstitial cystitis/bladder pain syndrome is a condition characterized by pain in the bladder region and by urinary urgency and increased urination frequency [30]. Current therapeutics are often insufficient for treating this condition, so the identification of new drug targets is of particular interest. Much like in animal models of neuropathic pain, animal models of bladder pain reveal TRPM8 to be pro-nociceptive. For instance, the use of a novel TRPM8 antagonist, AMTB, increased intercontraction intervals in a rodent model of overactive bladder syndrome, and also decreased the visceromotor reflex [31]. A similar phenomenon was observed in guinea pigs, as a novel TRPM8 antagonist reversed the reduction in bladder voiding volume induced by cold saline and menthol infusion in to the bladder [32]. Whether these effects are mediated via inhibition of TRPM8 channels on bladder-projecting afferents or on TRPM8 located in the bladder itself is unclear, as TRPM8 expression at the mRNA and protein levels has been observed in both afferents innervating the bladder [31,33,34] and in the bladder itself [34,35]. A recent study may shed some light on this, as recordings from C-fiber afferents demonstrated reduced firing in response to bladder distention in the presence of menthol when a novel TRPM8 antagonist was infused into the bladder [36]. Interestingly, increased TRPM8 immunostaining was observed in bladder samples from individuals with bladder pain syndrome, and this was moderately correlated with increased pain scores in those patients [34].

## 5. The Role of TRPM8 in Migraine

In addition to its role in somatic pain sensation, recent genome-wide association studies have found a significant correlation between migraine incidence and single nucleotide polymorphisms (SNPs) located near the TRPM8 coding region (for a review see [37]). Interestingly, this connection seems to be present only for individuals of Northern European ancestry [38,39,40,41,42], as studies involving populations from Spain, India, and China found either no association or weak associations between migraine incidence and SNPs near the *TRPM8* locus [43,44,45,46,47].

These human studies have naturally sparked interest in exploring the contribution of TRPM8 to migraine through the use of rodent models. Unfortunately, these studies have found opposing results concerning TRPM8’s involvement. Burgos-Vega and colleagues observed that application of icilin to the dura mater resulted in reduced paw and facial withdrawal thresholds in response to a mechanical stimulus, indicating that activation of TRPM8 caused migraine-like behaviors [48]. These behaviors were then subsequently blocked by dosing animals with a novel TRPM8 antagonist. Perhaps most interesting, however, was that sumatriptan, a drug commonly used to treat migraines, also prevented the migraine-like behaviors, which strongly implicates a role for TRPM8 in migraine generation. Conversely, a recent study by Ren and colleagues found opposing results, with application of menthol to the dura mater relieving migraine-like symptoms brought on by the application of inflammatory mediators to the dura. Symptom relief could then be subsequently blocked by injection of a TRPM8 antagonist. Finally, adding even more confusion to the matter is a study from Huang et al. that reported that TRPM8-expressing afferents were virtually absent from the dura [49]. However, further interest in strategies targeting TRP channels as migraine therapeutics is supported by clinical success of CGRP receptor antagonism since activation of TRP channels can elicit CGRP release [50].

## 6. Why is TRPM8 Analgesic in Some Cases and Nociceptive in Others?

An important point about TRPM8’s roles in nociception and analgesia is that activation of TRPM8 seems to consistently cause cold pain following injury, while simultaneously reducing mechanical and heat pain. Therefore, whether to target TRPM8 with either an agonist or antagonist may depend on which symptom is most troublesome to the patient; individuals with a primary complaint of mechanical hyperalgesia may respond best to TRPM8 agonists, while those with cold hyperalgesia may respond best to TRPM8 antagonists. Importantly, this is not to suggest that TRPM8 itself is sensitive to both mechanical and cold stimuli; indeed, afferent recordings indicate that pharmacological blockade of TRPM8 has no effect on baseline mechanical responsiveness [51] and genetic deletion of TRPM8 or pharmacological ablation of TRPM8-expressing afferents does not impact behavioral responses to mechanical stimuli [15]. Rather, it seems that the effects of TRPM8 agonism/antagonism are due to effects at the spinal level, with TRPM8-expressing afferents either directly or indirectly inhibiting mechanonociceptive afferents. Indeed, Proudfoot et al. reported that the analgesic effects of TRPM8 activation may be due to axoaxonic synapses on mechanonociceptive and heat-nociceptive afferent terminals, which contain inhibitory mGluRII and mGluRIII receptors [13]. Thus, release of glutamate from TRPM8-expressing afferents may decrease the amount of excitatory neurotransmitters released onto nociceptive projection neurons in lamina I and II of the dorsal horn. There is also a suggestion that topical menthol-induced pain relief may occur through blockade of voltage gated sodium channels [52].

At the same time, cold hyperalgesia following injury may be due to activation of a separate population of TRPM8-expressing nociceptors that relay painful information to the central nervous system. These nociceptors may have reduced thresholds for activation under injury conditions, leading to the observed strong hyperalgesic responses to cold. Additionally, they may be triggered by especially strong stimuli that facilitate TRPM8 activation; indeed, studies in humans consistently report that application of high concentrations of menthol (30%–40%) induces pain, cold allodynia, and cold hyperalgesia [53,54,55], whereas the lower concentrations used in topical agents induce analgesia.

## 7. Development of TRPM8 Antagonists for Chronic Pain

Antagonists of TRPM8 as therapeutics for chronic pain, migraine or inflammation have been pursued over the recent decade by many pharmaceutical companies such as Hydra Biosciences, Glenmark, Janssen, Pfizer, Bayer, Grunenthal, Mitsubishi Tanabe, RaQualia, Dompe/Axxam, BASF, Dendreon and Amgen. These efforts have led to the publication of many patents (for a review see [56,57,58]).

Janssen has published several potent and selective small molecule antagonists that suppresses icilin-induced wet-dog shakes, cold pressor response, as well as cold-induced allodynia in a neuropathic pain model in rats with a similar resulting dose-response range of effect in either of the cold-induced endpoints [59,60,61,62,63]. Glenmark also similarly reported efficacy with their antagonist in both wet-dog shakes and oxaliplatin-induced cold allodynia at the same 30 mg/kg dose [64]. RaQualia published RQ-00203078 which is a single digit nM antagonist at human or rat TRPM8 that also potently blocks icilin-induced wet-dog shakes in rats and is now commercially available, though evaluation in analgesic models has not been published [65].

AMG2850 is a ~200 nM potent and selective antagonist from Amgen that blocks both TRPM8 agonist-induced behavioral responses (wet-dog shakes) and cold-induced increases in blood pressure (cold pressor test)—both considered pharmacodynamic models of TRPM8 antagonism in vivo. Although the effect in cold allodynia was not evaluated, the effective dose would presumably be similar to that in the reported cold endpoint, the cold pressor test. There was, however, no evidence of meaningful reversal of inflammatory nor neuropathic-induced mechanical hypersensitivities in rats. This lack of efficacy occurred even at unbound plasma concentrations in excess of 21-fold the IC_90_ pharmacodynamic model suggesting that TRPM8 does not play a role in these mechanical pain behaviors at what would be reasonably considered more than enough target coverage [51,66,67], thus casting doubt on the therapeutic potential of TRPM8 as an analgesic in non-cold related conditions.

Pfizer did advance to clinical trials with a ~100 nM molecule, PF-05105679, which successfully inhibited the cold pressor response in humans, but also produced hot sensations that were both unexpected and considered adverse. The lack of therapeutic index to this event coupled with the short half-life in humans limited further clinical progression as well as the ability to evaluate the analgesic effect [32,68]. It has been demonstrated that cutaneous TRPM8 controls autonomic and behavioral thermoeffectors involved in body temperature maintenance with antagonists decreasing body temperature in rodents [26,69,70,71]. While the Pfizer antagonist did not produce a significant alteration of body temperature in healthy volunteers, further understanding is needed to elucidate whether and how these hot sensations may be on-target side effects in humans.

Chemotherapeutic drug-induced cold allodynia can be dose limiting, resulting in the cessation of treatment, enduring years beyond treatment, and for which there are currently no proven therapies [72,73,74]. Since chemotherapy is associated with changes in TRPM8 expression [25,75], perhaps TRPM8 antagonists could be beneficial in the prevention and/or reversal of this chemotherapy-induced cold allodynia. Or, perhaps TRPM8 antagonists could still be useful therapeutics for any other cold-related painful allodynia or hyperalgesia associated with other neuropathic or inflammatory conditions or even migraine or bladder pain. Currently, there are no ongoing trials with TRPM8 antagonists [76].
